# Clinical and Hematological Follow-Up of Long-Term Oral Therapy with Type-I Interferon in Cats Naturally Infected with Feline Leukemia Virus or Feline Immunodeficiency Virus

**DOI:** 10.3390/ani10091464

**Published:** 2020-08-20

**Authors:** Esperanza Gomez-Lucia, Victorio M. Collado, Guadalupe Miró, Sonsoles Martín, Laura Benítez, Ana Doménech

**Affiliations:** 1 Department of Animal Health, Veterinary Faculty, Complutense University of Madrid, 28040 Madrid, Spain; vmcollado@hotmail.es (V.M.C.); gmiro@ucm.es (G.M.); domenech@ucm.es (A.D.); 2 Department of Animal Medicine and Surgery, Veterinary Faculty, Complutense University of Madrid, 28040 Madrid, Spain; sonsolmi@ucm.es; 3 Department of Genetics, Physiology and Microbiology, Faculty of Biology, Complutense University of Madrid, José Antonio Novais, 12, 28040 Madrid, Spain; lbenitez@ucm.es

**Keywords:** FeLV, FIV, interferon, therapy, feline retrovirus, clinical outcome, immunomodulator, anemia

## Abstract

**Simple Summary:**

The viruses which produce feline leukemia (FeLV) and feline immunodeficiency (FIV) attack cells involved in the immune response. As specific drugs are either nonexistent, produce secondary effects, or are expensive, a therapeutic possibility is using nonspecific immunostimulants, such as human interferon alpha. We used this drug to treat 27 cats infected by FeLV and 31 infected by FIV. All cats were naturally infected and treatment was administered orally by their owners for four months. Participating cats were evaluated in our clinics at mid-treatment (M2), end of treatment (M4), and 4–8 months after the end of treatment (M10). We observed that treatment was well tolerated by the cats (as it did not affect the liver or the kidney functions), and improved most of the parameters analyzed (clinic, anemia, white cell counts, and CD4+/CD8+ ratio) as long as it was administered. However, 4–8 months after it was discontinued, though most animals remained clinically healthy, many of these parameters had rebounded to initial values or values even worse than the initial values. Thus, more studies should be conducted with longer administration of this drug to evaluate tolerability and sustained improvement of diseases produced by these two viruses which may lead to death.

**Abstract:**

Feline leukemia virus (FeLV) and feline immunodeficiency virus (FIV), two of the most important pathogens of cats, produce chronic systemic diseases with progressive death of cells involved in the immune response, ultimately leading to death. Immunostimulants is one of the few alternatives to the symptomatic treatment. In this study, 27 naturally FeLV-infected (FeLV+) and 31 naturally FIV-infected (FIV+) cats were administered orally by their owners 60 IU/day of recombinant human interferon alpha (rHuIFN-α) for four months in alternate weeks. Clinical status was evaluated and blood samples collected at four different visits or months (M): pretreatment (M0), mid-treatment (M2), end of treatment (M4), and 4–8 months after end of treatment (M10). Most cats ostensibly improved their clinical status, and many became asymptomatic. rHuIFN-α treatment improved the anemic processes observed at M0 (at least in cats with mild or moderate anemia) and leukocyte counts, including a more favorable CD4+/CD8+ ratio. An increase in the serum gammaglobulin concentration was seen in 80% of the cats. Despite observing an obvious favorable progress in the clinical, biopathological, and CD4+/CD8+ values during treatment, almost invariably all the parameters analyzed worsened after treatment discontinuation (M10), which suggests that the interferon-α protocol should be either extended or include additional cycles for a long-lasting benefit in FeLV+ and FIV+ cats.

## 1. Introduction

Feline leukemia and feline immunodeficiency viruses (FeLV and FIV, respectively) are retroviruses that affect domestic cats. Feline leukemia is a severe disease with several outcomes associated mainly with the immune response of the cat [1,2,3]. Disease outcomes range from “abortive infection” (a strong immune response eliminates the virus at the initial stages of the infection) to “regressive infection” (the virus remains integrated in the haemopoietic stem cells in the bone marrow) and “progressive infection” (when the virus is constantly expressed, associated to persistent viremia and progression of the FeLV infection to related diseases) [4,5]. Usually, FeLV-infected (FeLV+) cats die within 1 to 3 years after the initial diagnosis due to all the pathologies and clinical complications the cats develop [1], mostly produced by secondary infections that may arise because of immunodeficiency, but also to lymphoma and other neoplastic diseases. Infections by FIV progress following several phases similar to human immunodeficiency virus (HIV) infection in humans, leading in some cats to a final stage characterized by a progressive decline of CD4+ T lymphocytes that predisposes to many secondary infections and, eventually, to the death of the cat [1]. Naturally infected cats may remain apparently healthy for many years under adequate housing conditions with appropriate care and food [6], but recovery from FIV infection has never been documented.

As most clinical signs of both infections are associated with immunosuppression and secondary diseases, an approach to therapy is the use of immune modulators, such as interferon (IFN), which increase innate immunity and reduce viral replication and spread to target cells and organs [7]. Due to the antiviral and immunomodulatory effects of IFN-I, interferons have been employed for some time in the treatment of infectious diseases of small animals. Several published studies show its potential usefulness in feline medicine, including viral infections by herpesvirus, papillomavirus, coronavirus, parvovirus, and retroviruses [1,8,9,10,11,12]. As mentioned above, many FeLV- or FIV-infected cats suffer immunosuppression which allows the establishment of concurrent bacterial, viral, protozoal, and fungal infections [1]. The most common ones are *Toxoplasma gondii*, *Cryptococcus neoformans-Cryptococcus gattii* complex, *Haemobartonella felis*, *Mycoplasma haemofelis*, *Mycobacterium* spp., feline infectious peritonitis, feline panleukopenia, or feline calicivirus [1,5,13,14], but any other secondary or opportunistic pathogen can benefit from the immunosuppression. The incidence of this concomitant infections varies from region to region and with other factors such as age, outdoor access, etc. [1,15]. Presently, the use of more sensitive techniques allows the detection of subclinical infections which would otherwise pass unnoticed to the clinician [16] and which may be affected by different treatments. This detection is very difficult to apply in the routine practice, and the veterinary clinician is challenged by the need of treating retrovirus-infected cats not knowing if they have any other infection. Hence the interest of doing studies in the normal conditions the practitioner may encounter, in cats in which the moment of infection by FeLV or FIV is unknown and in which it is not possible to determine the multiple concomitant infections they may suffer. In any case, treatment with IFN-I may help the retrovirus-infected cat fight these pathogens.

IFN molecules used for therapy are produced by recombinant technology and include recombinant human IFN-α (rHuIFN-α) and recombinant feline IFN-ω (rFeIFN-ω). rHuIFN-α has the advantages over rFeIFN-ω, which is the only currently European Medicines Agency (EMA) licensed IFN for cats, of being active in different species, non-toxic, low-cost, and easy to administer orally [17]. In addition, it has been described to reduce in vitro the amount of FeLV and FIV infective particles in cell cultures [7].

Different protocols and routes of administration of rHuIFN-α have been evaluated. Neutralizing antibodies against rHuIFN-α are raised when it is administered parentally, ruling out treatment for more than 3–7 weeks [1,18], but not when administered orally for a longer time. Several studies reported the effect of rHuIFN-α, mainly in FeLV-infected cats (reviewed in [9,19]). In general, these studies associate the effect of this cytokine with decreased mortality [17], improvement in the clinical signs, laboratory alterations, CD4+/CD8+ ratio, and viral parameters of FeLV+ cats treated with IFN-α [18,20]. Very few studies describe results of treatment with rHuIFN-α in FIV infection, but they seem to be similar to those reported for FeLV infection, though no significant effects of rHuIFN-α were observed with respect to viral and proviral load [20,21]. The aim of the present study was to analyze the progress of the clinical status, biopathological markers, and CD4+/CD8+ ratio in FeLV- and FIV-naturally infected cats during oral treatment for four months with rHuIFN-α and several months after treatment discontinuation, as it is not known whether changes in these parameters are long-lasting. This study tries to reproduce the genuine conditions when treatment is administered by the cat owners at home and reflects the everyday activity of the veterinary clinician, in which when therapy is initiated, it is generally unknown when the animal became infected or whether there may be a subclinical infection by other pathogen. The results of the study suggest that rHuIFN-α has a very positive impact on the clinical status of the infected cats, as well as on the other parameters analyzed. However, this is not long-lasting and several months after treatment discontinuation several patients recede to the initial stage or worse.

## 2. Materials and Methods

### 2.1. Animals and Treatment

Fifty-eight cats were included in the study (27 FeLV+ and 31 FIV+). They lived with their owners, though most of them had been found stray before. Their age ranged between six months and 14 years, 31 were male (22 neutered), and 27 female (17 spayed). They were mostly of mixed breed (commonly known as domestic shorthair). They had been taken by their owners to one of the four participating private veterinary practices or to the Veterinary Clinical Hospital of the Complutense University of Madrid (VCH-UCM), either because they were ill, or for routine check-ups. Many of the cats had been collected recently from the street or adopted from a feline community. For this reason, the vaccination status was unknown in many cases. All cats enrolled tested positive to FeLV or FIV by the serologic Snap Combo Plus (Idexx Laboratories Inc., Westbrook, ME, USA), which detects FeLV p27CA and antibodies against FIV p24CA, and by a nested PCR [22]. They were all naturally infected and the exact moment of the infection was unknown. Presumably, they were in different stages of the disease, as asymptomatic cats and others with severe or mild disease were included in the study. Pregnant queens, aggressive cats, with neoplasia, in the terminal stages of the disease (complete lack of appetite and prostration and generalized lymphadenopathy), with other concomitant severe diseases or which had been diagnosed with feline infectious peritonitis, feline coronavirosis, feline herpesvirosis, or parasites were excluded from the study.

Owners signed an informed consent form for their cat to be included in the study, and agreed to administer the treatment completely and not to treat the cat with any other immunomodulator, but otherwise to care normally for the animal. Recombinant human IFN-α(2a) (rHuIFN-α, Roferon-A, Roche Diagnostics) was used, diluting the commercial vial with sterile saline solution to 60 IU/mL/dose, and storing the doses at 4 °C until use. The protocol proposed by Pedretti et al. [21] with slight modifications was followed. Owners were trained on how to administer treatment at home, consisting of one daily dose administered orally with food or directly in the mouth, in alternate weeks during four months, to a total of 56 doses. Owners were responsible for administering all doses, and for following the instructions precisely.

Animal handling, treatment, reagent manipulation, and data collection were all carried out in compliance with guidelines for Good Clinical Practice, and Good Laboratory Practice of the Animal Welfare Committee of the Veterinary Clinical Hospital and the Complutense University, and the experimental procedures were approved by the Institutional Animal Care and Use Committee of the Complutense University [20].

### 2.2. Clinical Evaluation and Blood Sampling

Owners were requested to take the cat to one of the four private veterinary clinics or to the HCV-UCM at four different moments or visits: M0, at the beginning of the treatment; M2, in mid treatment or month 2 (±15 days); M4, at the end of the treatment or month 4 (±15 days); M10, six months after finishing the treatment (±2 months). At each visit, the veterinarian in charge sampled the cat and rated in a report chart the 17 clinical signs most frequently observed in feline retrovirosis according to literature and to the experience of the participating practitioners to obtain a clinical score (CS) [4]. Clinical signs included could be rated as 0 (not present), 1 (mild), or 2 (severe). According to this CS at M0, cats were classified into three clinical groups (CG): CG1, with no clinical signs (asymptomatic); CG2, with a CS ≤ 5 (mild disease); and CG3, with a CS ≥ 6 (severe disease). Veterinarians had been instructed to report specifically if any cat had acquired any concomitant disease mentioned above.

Stressed cats were tranquilized with medetomidine (Domitor^®^, Pfizer Salud Animal SA, Madrid, Spain) for better management and to avoid the development of stress leukogram. Blood (2 mL) was collected from the cephalic or jugular veins and distributed into a tube with EDTA and another with heparin-lithium and sent immediately to the Department of Animal Health of the Veterinary Faculty of Madrid.

### 2.3. Biopathological Analyses and Determination of Anti-IFN-α Antibodies

The hemogram was determined automatically (Sysmex F-800 Microcellcounter, Sysmex Corp., Kobe, Japan) from EDTA anticoagulated blood. Differential leucocyte counts were determined manually as described previously [4]. Plasma urea, creatinine, and alanine aminotransferase (ALT) were determined with Reflotron (Boehringer-Mannheim, Mannheim, Germany) and total proteins by a refractometer (Atago T2, Co, Ltd. Tokyo, Japan). The electrophoretogram was performed as described previously [23]. The reference values used for normality were enumerated previously [4] and are also shown in Tables S2 and S3 in the Supplementary Files. A commercial ELISA test (Bender MedSystemsTM, Vienna, Austria) was used to detect the development of anti-IFN-α antibodies in serum during treatment.

### 2.4. Evaluation of the CD4+/CD8+ Ratio

The method described by Collado et al. [4] was followed to determine the CD4+/CD8+ ratio in the EDTA-anticoagulated blood using monoclonal antibodies against feline CD4 and CD8 labeled with fluorescein and rodamin (Southern Biotechnology Associates Inc., Birmingham, AL, USA), respectively. Samples were analyzed in a flow cytometer (Becton Dickinson FAC-Scan, Becton Dickinson Biosciences, San Jose, CA, USA) in the Research Support Centre (CAI) of the UCM. The ratio was considered decreased when it was <0.9.

### 2.5. Statistical Analysis

The results obtained were analyzed in the Data Processing Center of the UCM using Origin Pro 7.5, SPSS 25.0 (IBM Corp. Released 2017. IBM SPSS Statistics for Windows, Version 25.0., Armonk, NY, USA) and Statgraphics Centurion XVIII (Statgraphics Technologies, The Plains, VA, USA, www.statgraphics.com). Multiple variable analyses were done. Results were compared using cross-tabulation, contingency coefficient, Fishers exact test, Chi-square, and Student’s *t*-test, with a significance of 0.95. Correlations ≥0.7 were considered high, and moderate when they were 0.4–0.7. All data were cross-tabulated. Only significant differences are mentioned in the text. However, when the number of cats in the groups to compare was not large enough to reach a statistically significant conclusion at 0.95, a significance of <0.8 was established to compare those data.

Data collected at each visit (M2, M4, and M10) were compared to the situation just before treatment was initiated (M0). Due to different circumstances, some owners did not take their cat to all visits. Thus, the statistical analysis of each visit has been performed with those that were taken and not with the whole study population.

## 3. Results

### 3.1. Epidemiological Data and Clinical Evolution of Cats Treated with rHuIFN-α

Within the FeLV+ cats there were more female (59.3%) than male cats (40.7%), while in the FIV+ cats more male (64.5%) than female cats (35.5%) were enrolled in the study (Table 1). The percentage of neutered or spayed cats infected by each virus was similar (~2/3). Most of the cats (87.9%) were common shorthair (known also as mixed breed). The study included naturally infected cats with different clinical status, possibly reflecting diverse stages of the disease. The clinical signs observed more frequently when cats were introduced in the study (M0) were loss of appetite, asthenia and prostration, altered mucosae, membranes, oral lesions, respiratory disorders, and lymphadenomegaly. However, approximately one-third of the cats were asymptomatic (Table 1). The laboratory findings and viral parameters in this group of cats at M0 is published elsewhere [4,20,23].

Though owners had been instructed to bring their cat to all three visits, for different reasons several failed in this obligation and some data were missing. Most owners took their cats while treatment lasted (high fidelity during M2 and M4), but only 44.4% of the FeLV+ and 77.4% of the FIV+ cats were taken to M10 (Supplementary Table S1). Only cats which had been brought to at least two of the three visits were included in the study.

**Figure 1:**
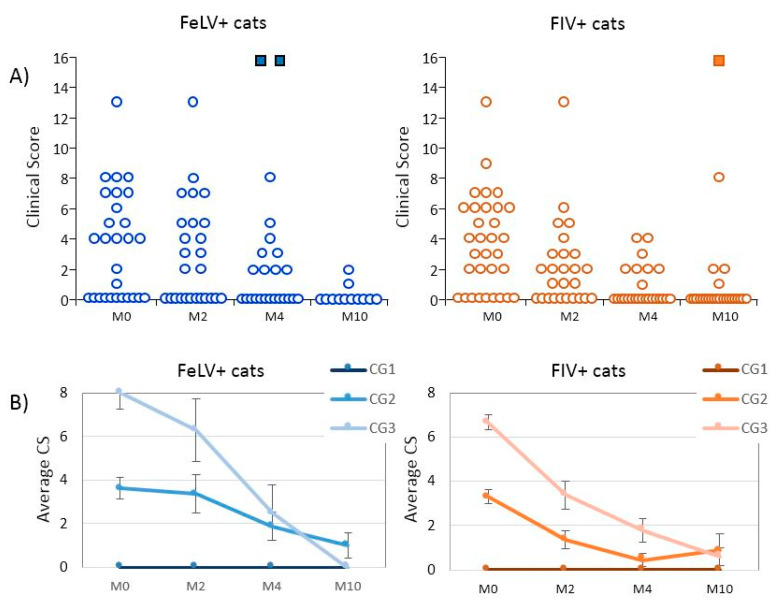
Evolution of the clinical score (CS) of the cats during the study. (**A**) General representation of the evolution. Each circle represents a cat analyzed, and each square a dead cat. (**B**) General trend of the average CS of the three clinical groups (CG) in which the cats were classified. Bars indicate standard error. M0, beginning of treatment; M2, two months (±15 days) after beginning of treatment (mid-treatment); M4, four months (±15 days) after beginning of treatment (end of treatment); M10, 10 months (±2 months) after beginning of treatment. CG1, asymptomatic cats; CG2, cats with mild disease; CG3, cats with severe disease.

Three cats died during the treatment period (2 FeLV+ and 1 FIV+ cats). All these three cats had a CS ≥ 6 at M0 (CG3). The two FeLV+ cats died between M2 and M4; the FIV+ cat died between M4 and M10, after having improved the CS between M0 and M4. Most cats (15 of the 16 FeLV+ sick cats; 20 of the 22 FIV+ sick cats) improved clinically when compared to M0 (Table 2; Table S1; Figure 1). None of the CG1 cats showed any clinical signs at any of the visits (enlisted under “stable evolution” in Table 2).

The clinical improvement encompassed all clinical signs initially observed. However, lymphadenomegaly and digestive disorders in FeLV+ cats were the most persistent signs and were still present in 8.3% of these cats at M10. In one FIV+ cat, the clinical condition was worse at M10 than at M4 (Table 3). No statistical differences were observed in clinical evolution with respect to the CG, sex or age.

### 3.2. Hemogram

Even CG1 (asymptomatic) cats had abnormalities in the hemogram. While treatment lasted there was a general improvement of the three hematological values studied, especially in packed cell volume (PCV) and hemoglobin concentration (Hgb), which had a parallel evolution, somewhat independent from the red blood cell counts (RBC). The data of all the analysis of each individual cat are shown in Supplementary Tables S2 and S3.

**Figure 2:**
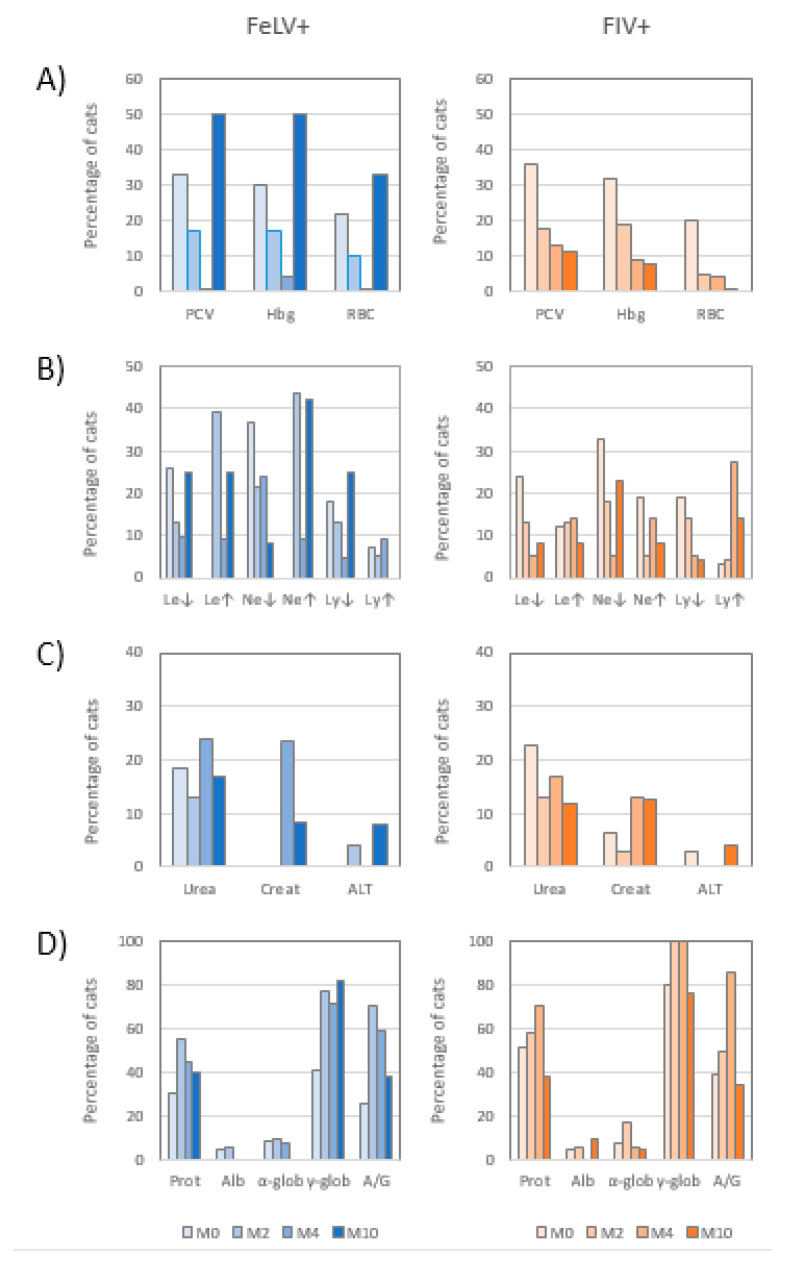
Percentage of FeLV+ and FIV+ cats treated with rHuIFN-α with altered hemogram (**A**), leukogram (**B**), biochemical profile (**C**), and electrophoretogram (**D**), at each time point of the study (M0, M2, M4, and M10). Htc, hematocrit; Hgb, hemoglobin concentration; RBC, red blood cells count. Le↓, leukopenia. Le↑, leukocytosis. Ne↓, neutropenia. Ne↑, neutrophilia. Ly↓, lymphopenia. Ly↑, lymphocytosis. Creat., creatinine. Prot., hyperproteinemia. Alb., hypoalbuminemia. α-glob, hyperalfaglobulinemia. γ-glob, hypergammaglobulinemia. A/G, disproteinemia or decreased albumin/globulin ratio. M0, beginning of treatment. M2, two months (±15 days) after beginning of treatment (mid-treatment). M4, four months (±15 days) after beginning of treatment (end of treatment). M10, 10 months (±2 months) after beginning of treatment.

The percentage of cats with altered hemogram decreased with treatment in both infections (Figure 2A). In FeLV+ cats the highest recovery was observed at the end of interferon treatment (M4), when the hemogram values were considered normal in most cats. However, the percentage of anemic cats increased when treatment was discontinued, following a “rebound pattern”. On the contrary, there was a constant decrease of the percentage of anemic FIV+ cats, and the lowest percentage was observed at M10. Differences in hemogram values between FIV and FeLV infections were significant at M10 (*p* < 0.05), but otherwise differences between sex, age, or clinical groups were not statistically significant. Four FeLV+ and six FIV+ cats with normal hemogram at M0 developed hemogram alterations during the study. With respect to the cats with abnormal hemogram values at M0, these values were normal at M10 in all but one FeLV+ and one FIV+ cats.

### 3.3. Leukogram

As both FeLV and FIV have been recognized as able to infect leukocytes, it is important to determine how the different populations of white blood cell (WBC) progress. Abnormalities in the absolute neutrophil and/or lymphocyte counts were detected at M0 in 45.2% of the FeLV+ and 55.3% of the FIV+ cats, when compared to the reference ranges used in the laboratory. Neutropenia was the most frequent alteration (35.5% in FeLV+ and 31.6% in FIV+ cats), and, in general, in both infections there was a higher percentage of low cell counts (cytopenias) than high cell counts (cytophilias). A high percentage (40.0%) of the FeLV+ cats with altered WBC counts had both the neutrophil and lymphocyte counts altered.

The percentage of cytopenic FeLV+ cats decreased during treatment. At M4, the percentage of FeLV+ cats with leukopenia was 9.5%, and with lymphopenia 4.8%. However, at M10 the “rebound pattern” mentioned above was observed for both values. By contrast, neutropenia was not detected to “rebound”, as only 8.3% of the FeLV+ cats were seen to have this condition at M10, compared to ~20% at M2 and M4 (Figure 2B). Neutrophilia developed in several cats at M2, and as a result, leukocytosis was present. Both leukocytosis and neutrophilia were low at the end of treatment (M4), but became high again when treatment was discontinued.

The leukogram in FIV+ cats treated with rHuIFN-α was characterized by the decrease of the percentage of cytopenic cats. However, the percentage of neutropenic cats increased at M10 (Figure 2B). Contrarily to FeLV+ cats, at M0 a few FIV+ cats had leukocytosis and/or neutrophilia, but the percentage of cats with neutrophilia was decreased at M2, though the percentage of both neutrophilia and lymphocytosis was increased at M4.

Blood smears did not evidence any signs of neoplasia that could be related to neutrophilia or lymphocytosis in any of the cats. Approximately half of the cats with neutrophilia (47.1% of FeLV+ and 50.0% FIV+ cats) had clinical signs possibly associated to secondary infections, such as lymphadenomegalia, conjunctivitis, oral or skin lesions, or respiratory disease.

No statistically significant differences were observed in the evolution of the biochemical profile with respect to sex, age, or clinical group.

### 3.4. Biochemical Profile

Biochemical parameters analyzed in plasma (urea, creatinine, ALT) were not greatly affected by retroviral infection in most infected cats. Only three FeLV+ cats had high ALT levels during the study (at M2 and M10). A higher percentage of cats (13–24%) had altered urea levels during the different visits. With respect to creatinine, the number of cats which had this parameter altered increased during treatment, especially in FeLV+ cats, none of which had azotemia at M0, but decreased after treatment discontinuation (Figure 2C). No statistically significant differences were observed in the evolution of the biochemical profile with respect to sex, age, or clinical group.

### 3.5. Electrophoretogram

Hypergammaglobulinemia was a common finding in both infections. At M0, 40.7% of FeLV+ cats and 80.6% of FIV+ cats had this condition (Figure 2D). Hypergammaglobulinemia influenced the concentration of total proteins and the ratio albumin/globulin (A/G). In FeLV infection, ~80% of the FeLV+ cats with normal serum gammaglobulin concentration at M0 developed hypergammaglobulinemia during treatment. The highest increase in serum gammaglobulins and total proteins in FeLV+ cats was observed at M2, and these alterations continued mostly throughout the study.

With respect to FIV+ cats, the percentage of cats with hypergammaglobulinemia increased during treatment, and 100% of FIV+ cats were hypergammaglobulinemic at M2 and M4. The percentages of FIV+ cats with hyperproteinemia, hypergammaglobulinemia, and dysproteinemia (altered A/G ratio) at M10 were similar or lower than those registered at M0 before starting treatment (37.5%, 77.3%, and 34.8%, respectively) (Figure 2D). Only three FeLV+ and three FIV+ cats were able to return to normal gammagloblin serum concentration during the study.

In both infections, high urea and/or creatinine concentrations always coincided with high gammaglobulinemia concentration. However, hypergammaglobulinemia was not always parallel to high urea and/or creatinine concentrations. In FeLV+ cats, the decrease in gammaglobulins and plasma urea was associated (*p* < 0.05).

### 3.6. Evolution of the CD4+/CD8+ Ratio

In both infections, the average value of the CD4+/CD8+ ratio increased with interferon treatment (Table 4). In FeLV+ cats, the highest average value was registered at M10, while in FIV+ cats it was at M4. The percentage of cats with subnormal CD4+/CD8+ ratio (<0.9) also decreased throughout treatment with interferon (Table 4). In FIV+ cats, the highest increase in the ratio was observed at M2, but treatment discontinuation involved lower values of the CD4+/CD8+ ratio in FIV+ cats.

### 3.7. Detection of Antibodies Anti-Interferon α by ELISA

Regardless of the human origin of the treatment, antibodies anti IFN-α were never detected in any of the cats treated orally with rHuIFN-α by the ELISA used.

## 4. Discussion

Feline leukemia and feline immunodeficiency are chronic systemic diseases, for which there is no specific treatment, and immunotherapy is a way to control the progress of both infections. Initially, rHuIFN-α began to be used in cats infected with FeLV or FIV [17] after the good results obtained in the treatment of HIV infection [24] and by the absence of a similar molecule of feline origin. When rFeIFN-ω became available, it largely displaced the application in veterinary medicine of rHuIFN-α. However, rHuIFN-α has as advantages over rFeIFN-ω that the oral administration of rHuIFN-α facilitates the continuity of treatment as it can be done by the owner; and the low concentrations of rHuIFN-α required reduce significantly the cost of treatment of these chronic diseases. However, there are few studies on the effect of rHuIFN-α treatment on FeLV or FIV infected cats in a real situation, as most studies are conducted on experimentally infected cats and in controlled environments. Therefore, it can be difficult to ensure the result that is expected to be obtained when applying the treatment under conditions of natural infection, without knowing the current situation of the cat infection and if the owner will follow exactly the protocol at home.

In this study, we followed-up the disease in each cat by comparison to M0, before the start of the treatment. This approach has been followed by other researchers when studying naturally infected cats [20,25,26,27,28], who have suggested that values at M0 are potentially more representative than a placebo in studies in field conditions with animals in unknown and probably different stages of infection. In addition, a placebo-treated or non-treated group of cats would have been ethically controversial, as owners demand a therapeutic option for their cats.

Our results show that FIV+ and FeLV+ cats treated with rHuIFN-α improved greatly their clinical status as well as many of the biopathological parameters studied, similar to that described previously with rFeIFN-ω [10,25,28,29]. The improved clinical situation of the vast majority of cats was maintained over time in the absence of treatment. No cat with asymptomatic disease at M0 (CG1) developed clinical signs during treatment; this may indicate that rHuIFN-α is also valuable in delaying or avoiding disease in infected cats.

The hemogram is one of the most important factors to determine the clinical situation of a sick cat and is used to predict the evolution of the infection in FeLV+ cats [10,30]. An improvement of the hemogram was observed in FeLV+ or FIV+ cats treated with rHuIFN-α. This agrees with that described by other authors for cats treated with rHuIFN-α [21] or with rFeIFN-ω [10,28], but not with others [25,29,31,32,33]. Disagreement may be due to variable FeLV virulence or initial severity of anemia in treated cats. Decreased replication of FeLV in erythrocyte precursors and stroma cells may account for the improvement of the hemogram in FeLV+ cats treated with rHuIFN-α. In our study, the response and progress of the hemogram was different in FeLV+ and FIV+ cats. In FeLV+ cats we observed what we termed “rebound pattern”, as most hematological results were worse at M10 than at M4. Contrarily, most FIV+ cats improved their hematological parameters at M10. We also observed the “rebound pattern” in the viral follow-up in these same cats, especially in FeLV+ cats [20], suggesting that rHuIFN-α only controls the effects produced by the virus as long as it is administered.

The percentage of FeLV+ or FIV+ cats with cytopenias decreased transiently at M2 and M4 of treatment with rHuIFN-α, when compared to M0. This agrees with results of cats treated with rFeIFN-ω [28,29] but had not been reported previously in rHuIFN-α-treated cats. It may mean that rHuIFN-α stimulates the regeneration of white blood cells, as a result from either decreasing the rate of replication of the viruses, or stimulating the proliferation of lymphocytes. In any case, an improved leukogram would mean a better immune response and, consequently, an improvement of the clinical situation. The “rebound pattern” after treatment discontinuation was observed in both FeLV and FIV infections, which had also been described by Pedretti et al. [21].

Our results show that treatment with rHuIFN-α at the concentration, length of time and route of administration used did not affect the liver function in most cats, similar to results by other authors [25,31,32,34]. As respects the renal parameters, all cats which developed high urea and/or creatinine values had hypergammaglobulinemia. In our study, most treated cats developed hypergammaglobulinemia which may result from an increased polyclonal activation of B cells [35,36] in response to the immune stimulation by IFN-α. However, in FeLV+ and FIV+ cats the progress of the hypergammaglobulinemia was different, which suggests a different mechanism in both infections. FeLV+ cats responded very fast to treatment, as almost 80% of them had become hypergammaglobulinemic at M2, a percentage that remained stable even at M10, twice as high as in M0. The average concentration of gammaglobulins in FIV+ cats was higher than the corresponding one in FeLV+ cats at all time points, and hypergammaglobulinemia has been observed to be one of the characteristics of FIV infection [23]. In FIV+ cats, the highest percentage of hypergammaglobulinemia was seen at M4 and decreased at M10.

FIV-infected cats usually have a decreased CD4+/CD8+ ratio [5]. Our results of M0 support this observation and associate the ratio to the clinical situation [4]. In both infections, the CD4+/CD8+ ratio and the percentage of cats with normal ratio increased when they were treated with rHuIFN-α, contrarily to studies by others [21,37]. In FeLV+ cats, the positive effect of rHuIFN-α was more evident than in FIV+ cats, and lasted throughout the study, unaffected by the “rebound pattern” mentioned above. In FIV+ cats, the ratio increased faster but also decreased as soon as treatment ended, though at M10 the values were still ≥0.9 (normal) in 65% of the animals (vs. 30.8% at M0). According to our results [28], rHuIFN-α is more effective than rFeIFN-ω in improving the CD4+/CD8+ ratio.

The results shown here follow a similar trend to those of the viral parameters, including antigenemia (p27) and RT activity and proviral load in FeLV+ cats [20], in the sense that most of these values that were seen to improve during treatment rebounded into unfavorable values after treatment was discontinued. This may mean that the worsening of the viral condition in FeLV-infected cats may imply a deterioration in the biopathological parameters with little consequences on the clinical status of the cat. However, rHuIFN-α did not affect viral parameters of FIV+ cats.

One of the limitations of the present study is that concomitant diseases were not thoroughly diagnosed in the animals included. In the selection and follow-up of the subjects of our study we relied on the expertise of the collaborating veterinary practitioners, some of them with over 30 years of experience, who presumably would have detected accompanying illnesses, and act in consequence. However, according to a study with blood donors [16], even asymptomatic cats may be infected by any of the innumerable pathogens of cats, which may have gone undetected in our study. As in the literature reviewed, IFN-α had been studied only for treating herpesvirus [38], enteroviruses [10], parvovirus [11], and coronavirus [12] in cats, it is unclear whether if coinfection had occurred how would it have progressed. However, the mechanism of action of this cytokine is quite nonspecific and may have improved the clinical outcomes in other infections (for a review on the mechanism of action see in [8]). Regardless of the limitation of the potential concomitant diseases, the study presented here has the value of representing the reality faced by the clinician when treating FeLV- or FIV-infected patients, as most frequently these cats are not tested for other pathogens, unless they present a clinical disease compatible with other infections.

## 5. Conclusions

When used in FeLV or FIV naturally infected cats, rHuIFN-α may improve the clinical status; increase the RBC counts; decrease the anemia; increase the neutrophil and lymphocyte counts, improving the immune response against the virus and secondary infections; and improve the CD4+/CD8+ ratio.

The reversion of several of the factors studied to initial values after treatment was withdrawn suggests that treatment with rHuIFN-α was not as long as necessary or may require repeated cycles to consolidate the improvement.

The clinical, biopathological, and CD4+/CD8+ ratio, along with the viral follow-up described previously [20], suggest that rHuIFN-α administered orally by the cat owner is an interesting option for treating FeLV- or FIV-infected cats.

## Figures and Tables

**Table 1:** Epidemiological data of the cats enrolled in the study and distribution of the clinical groups (CG). CS, clinical score.

	FeLV+ (n = 27)	FIV+ (n = 31)
Age in years: mean (range)	1.9 (0.3–6)	4.8 (0.6–14)
Sex: males/females	11/16	20/11
Neutered: yes/no	18/9	21/10
Breed: mixed breed/(breed)	25/2 (Persian)	26/5 (2 Persian, 2 Siamese, 1 Bombay)
CG1 (CS = 0; asymptomatic)	11 (40.7%)	9 (29.0%)
CG2 (CS: 1–5; mild disease)	8 (29.6%)	13 (41.9%)
CG3 (CS: ≥6; severe disease)	8 (29.6%)	9 (29.0%)

**Table 2:** Evolution at each visit of the clinical score (CS) of infected sick cats (CG2 and CG3) treated with rHuIFN-α compared to the beginning of treatment (M0).

Infection	Evolution	M2 n (%)	M4 n (%)	M10 n (%)
FeLV^+^	favorable	7 (43.8%)	13 (81.2%)	5 (100%)
	stable	8 (50.0%)	0	0
	unfavorable	1 (6.2%)	3 ^a^ (18.8%)	0
FIV^+^	favorable	17 (89.4%)	18 (100%)	15 (88.2%)
	stable	1 (5.3%)	0	1 ^b^ (5.9%)
	unfavorable	1 (5.3%)	0	1 ^a^ (5.9%)

M2, month 2 ± 15 days; M4, month 4 ± 15 days; M10, month 10 ± 2 months; favorable, decrease in the CS; unfavorable, increase in the CS; stable, the same CS. ^a^ Two FeLV+ and one FIV+ cats were reported dead at this visit. ^b^ One FIV+ cat had higher CS at M10 than at M4.

**Table 3:** Percentage in which the different clinical signs in FeLV+ and FIV+ cats treated with rHuIFN-α were observed. M0, beginning of treatment; M2, two months (±15 days) after beginning of treatment (mid-treatment); M4, four months (±15 days) after beginning of treatment (end of treatment); M10, 10 months (±2 months) after beginning of treatment.

	FeLV				FIV			
	M0 (n = 27)	M2 (n = 26)	M4 (n = 22)	M10 (n = 12)	M0 (n = 31)	M2 (n = 25)	M4 (n = 25)	M10 (n = 24)
Loss of appetite	33.3	32.0	13.6	0.0	54.8	25.0	11.5	4.2
Asthenia	33.3	28.0	9.1	0.0	41.9	29.2	15.4	4.2
Body condition	18.5	4.0	4.5	0.0	19.4	8.3	0.0	4.2
Lymphadenomegaly	18.5	16.0	13.6	8.3	19.4	12.5	7.7	0.0
Altered mucosae	25.9	12.0	4.5	0.0	29.0	4.2	0.0	4.2
Conjunctivitis	25.9	24.0	13.6	0.0	16.1	12.5	7.7	4.2
Oral lesions	29.6	28.0	22.7	0.0	32.3	20.8	3.8	4.2
Digestive disorders	11.1	4.0	0.0	8.3	9.7	8.3	0.0	4.2
Skin lesions	11.1	8.0	0.0	0.0	12.9	4.2	3.8	0.0
Respiratory disease	29.6	24.0	9.1	0.0	22.6	4.2	7.7	8.3

**Table 4:** Average value of the CD4+/CD8+ ratio (normal value ≥0.9), and percentage of cats treated with rHuIFN-α with subnormal ratio.

	Infection	M0	M2	M4	M10
Average CD4+/CD8+ ratio	FeLV^+^	1.66	1.79	2.11	2.38
	FIV^+^	0.80	1.17	1.40	1.29
Percentage of cats with altered CD4+/CD8+ ratio (<0.9)	FeLV^+^	26.1%	10.0%	5.3%	0%
	FIV^+^	69.2%	30.0%	20.0%	35.0%

M0, beginning of treatment. M2, two months (±15 days) after beginning of treatment (mid-treatment). M4, four months (±15 days) after beginning of treatment (end of treatment). M10, 10 months (±2 months) after beginning of treatment.

## Supplementary Materials

The following are available online at https://www.mdpi.com/2076-2615/10/9/1464/s1, Table S1: Follow-up of the clinical score (CS) of each individual cat infected either by FeLV or by FIV belonging to each clinical group (CG1, GG2 and CG3) at each moment of treatment with rHu-IFN. Table S2. Individual results of the FeLV-infected cats analyzed in the study. Table S3. Individual results of the FIV-infected cats analyzed in the study.
