# Supplementary material for: Clinical and Hematological Follow-Up of Long-Term Oral Therapy with Type-I Interferon in Cats Naturally Infected with Feline Leukemia Virus or Feline Immunodeficiency Virus
Source: Animals (Basel). 2020 Aug 20;10(9):1464. doi: 10.3390/ani10091464 (PMC7552327; doi:10.3390/ani10091464)
Supplement: Supplementary file 1 [file animals-10-01464-s001.zip › Table S2 v2.docx]

**Table S2**. Individual results of the FeLV-infected cats analyzed in the study. ID, identification of each cat. M, month of the visit as follows: M0, beginning of treatment. M2, two months (±15 days) after beginning of treatment (mid-treatment). M4, four months (±15 days) after beginning of treatment (end of treatment). M10, 10 months (±2 months) after beginning of treatment. PCV, packed cell volume (reference range 26-45%). Hgb, hemoglobin concentration (reference range 8-15 g/dL). RBC, red blood cell count (reference range 5-10 x 10^6^ cells/µL). Leuk, leukocyte count (reference range 5.5-19.5 x 10^3^ cells/µL). Ntr, segmented neutrophil count (reference range 2.5-12.5 x10^3^ cells/µL). Lym, lymphocyte count (reference range 1.5-7.7 x10^3^ cells/µL). Urea (reference range 38-71 mg/dL). Creat, creatinine (reference range 0.7-1.8 mg/dL). ALT, alanine amino transferase (reference range 30-100 UI/L). Prot, total proteins (reference range 5.7-7.9 g/dL). Alb, albumin (reference range 2.3-4.0 g/dL). α-s, serum α-globulins (reference range 0.5-1.5 g/dL). γ-s, γ-globulins (reference range 0.5-1.5 g/dL). A/G, albumin/globulins ratio (reference range 0.7-1.3). p27, concentration of the protein p27 of FeLV (mg/µl) by PetChek FeLV Antigen Test (Idexx). RT, reverse transcriptase activity (mU/mL) by C-type RT Activity Assay (Cavidi). CD4/CD8, CD4:CD8 ratio. rtPCR, real time PCR. Blank cells indicate that the analysis could not be done at that time point.

| **id** | **M** | **PCV** | **Hgb** | **RBC x 10^6^** | **Leuk x 10^3^** | **Ntr x 10^3^** | **Lym x 10^3^** | **Urea** | **Creat** | **ALT** | **Prot** | **Alb** | **α-s** | **γ-s** | **A/G** | **p27** | **RT** | **CD4**  **CD8** | **rt**  **PCR** |
| --- | --- | --- | --- | --- | --- | --- | --- | --- | --- | --- | --- | --- | --- | --- | --- | --- | --- | --- | --- |
| **FeLV-01** | 0 | 15 | 4.8 | 4.34 | 11.40 | 10.37 | 0.34 | 82 | 0.5 | 18 | 10.4 | 1.89 | 1.70 | 6.12 | 0.22 | 0.63 | 6.48 | 0.72 | 0.53 |
| **FeLV-01** | 2 | 19 | 5.10 | 4.52 | 10.00 | 9.70 | 0.23 | 76 | 1.2 | 11 | 9.0 | 1.91 | 1.41 | 4.09 | 0.27 | 0.95 | 5.98 | 1.07 | 0.43 |
| **FeLV-02** | 0 | 41 | 13.5 | 9.12 | 9.00 | 6.21 | 2.34 | 65 | 1.2 | 19 | 6.8 | 4.06 | 0.95 | 1.34 | 1.48 | 0.12 | 1.91 | 1.03 | 0.67 |
| **FeLV-02** | 2 | 32 | 10.3 | 6.99 | 19.00 | 10.07 | 6.65 | 61 | 1.1 | 22 | 6.6 | 3.52 | 0.84 | 1.48 | 1.14 | 0.17 | 2.24 | 1.15 | 0.79 |
| **FeLV-02** | 4 | 35 | 10.6 | 7.97 | 10.00 | 5.40 | 2.70 | 58 | 1.3 | 28 | 7.4 | 4.12 | 1.14 | 1.44 | 1.26 | 0.11 | 0.95 |  | 0.72 |
| **FeLV-03** | 0 | 30 | 9.8 | 5.55 | 1.50 | 0.03 | 1.47 | 48 | 0.8 | 30 | 6.2 | 2.78 | 1.37 | 1.38 | 0.81 | 1.58 | 0.75 | 0.45 | 0.49 |
| **FeLV-03** | 2 | 33 | 10.3 | 6.53 | 20.40 | 16.73 | 3.06 | 50 | 0.7 | 20 | 8.4 | 3.22 | 0.99 | 3.34 | 0.62 | 0.96 | 0.00 | 1.26 | 0.88 |
| **FeLV-03** | 4 | 34 | 9.60 | 6.70 | 18.40 | 12.38 | 3.52 | 50 | 0.8 | 21 | 7.6 | 3.23 | 1.09 | 3.15 | 0.70 | 0.04 | 0.00 | 2.15 | 0.69 |
| **FeLV-04** | 0 | 45 | 15.7 | 10.54 | 12.80 | 8.06 | 3.20 | 42 | 1.1 | 13 | 8.8 | 3.54 | 1.29 | 3.40 | 0.67 | 0.64 | 3.95 | 1.28 | 0.39 |
| **FeLV-04** | 2 |  |  |  |  |  |  | 57 | 1.6 | 17 | 7.2 | 2.63 | 1.53 | 2.11 | 0.58 | 0.18 | 0.00 |  | 0.49 |
| **FeLV-04** | 4 | 38 | 14.3 | 9.80 | 15.30 | 3.96 | 9.71 |  |  |  |  |  |  |  |  | 0.13 | 0.00 | 1.97 | 0.48 |
| **FeLV-05** | 0 | 32 | 11.1 | 7.88 | 9.70 | 6.80 | 3.60 | 43 | 1.2 | 16 | 8.0 | 3.21 | 1.23 | 2.92 | 0.67 | 1.19 | 0.35 | 1.48 | 0.54 |
| **FeLV-05** | 2 | 44 | 13.5 | 9.80 | 28.50 | 21.66 | 5.70 | 46 | 1.1 | 19 | 8.0 | 3.32 | 1.15 | 2.69 | 0.71 | 0.45 | 0.00 | 1.56 | 0.64 |
| **FeLV-05** | 4 | 35 | 10.5 | 6.59 | 3.70 | 0.33 | 2.37 | 76 | 2.0 | 31 | 8.2 | 3.15 | 1.13 | 2.97 | 0.62 | 0.07 | 0.00 | 1.71 | 0.47 |
| **FeLV-06** | 0 | 42 | 14.8 | 10.02 | 0.01 | 7.20 | 4.50 | 76 | 1.4 | 47 | 9.4 | 3.25 | 1.12 | 4.52 | 0.53 | 0.40 | 6.44 |  | 0.76 |
| **FeLV-06** | 2 | 28 | 8.5 | 6.08 | 49.80 | 38.84 | 8.47 | 54 | 0.9 | 51 | 7.8 | 2.83 | 1.37 | 2.97 | 0.57 | 0.33 | 4.35 | 1.96 | 1.01 |
| **FeLV-06** | 4 | 47 | 15 | 11.60 | 50.20 | 39.49 | 9.91 |  |  |  | 8.2 | 3.02 | 1.15 | 2.91 | 0.58 | 0.07 | 0.00 | 1.77 | 0.83 |
| **FeLV-07** | 0 | 16 | 4.50 | 5.45 | 0.00 | 1.35 | 1.26 | 53 | 1.2 | 35 | 6.2 | 3.50 | 0.81 | 1.26 | 1.34 | 0.94 | 0.03 | 2.15 | 0.59 |
| **FeLV-07** | 2 |  |  |  |  |  |  | 57 | 0.8 | 109 | 5.8 | 2.79 | 1.37 | 0.97 | 0.92 | 0.48 | 0.00 |  | 0.52 |
| **FeLV-07** | 4 | 38 | 11 | 8.53 | 12.30 | 2.90 | 7.30 | 69 | 1.2 | 27 | 7.2 | 3.12 | 1.22 | 2.02 | 0.76 | 0.33 | 0.00 | 2.29 | 0.46 |
| **FeLV-07** | 10 | 30 | 9.2 |  | 25.90 | 19.68 | 4.14 | 51 | 1.2 | 13 | 7.8 | 3.42 | 1.30 | 1.83 | 0.78 | 1.02 | 0.03 | 1.93 | 0.69 |
| **FeLV-08** | 0 | 42 | 13.8 | 8.36 | 10.00 | 6.70 | 2.40 | 87 | 1.4 | 32 | 8.4 | 3.24 | 1.14 | 3.57 | 0.63 | 0.54 | 0.00 | 2.2 | 0.67 |
| **FeLV-08** | 2 | 38 | 12.4 | 7.53 | 13.80 | 4.51 | 7.46 | 44 | 1.3 | 23 | 8.6 | 3.56 | 0.88 | 3.39 | 0.70 | 0.07 | 0.00 | 3.25 | 0.89 |
| **FeLV-08** | 4 | 32 | 10 | 7.16 | 13.10 | 5.11 | 6.94 | 67 | 1.1 | 20 | 6.9 | 3.96 | 1.29 | 1.47 | 1.35 | 0.15 | 0.00 | 2.86 | 0.55 |
| **FeLV-08** | 10 | 18 | 5.6 | 3.67 | 18.70 | 16.46 | 0.56 | 110 | 1.5 | 135 | 7.8 | 3.03 | 1.39 | 2.59 | 0.64 | 0.67 | 9.88 | 2.11 | 0.76 |
| **FeLV-09** | 0 | 23 | 7.6 | 4.44 | 4.50 | 2.88 | 1.31 | 70 | 0.9 | 17 | 6.2 | 2.77 | 1.03 | 1.81 | 0.81 | 0.75 | 57.26 | 0.86 | 0.57 |
| **FeLV-09** | 2 | 30 | 9.9 | 9.33 | 27.10 | 21.68 | 3.52 | 47 | 0.6 | 25 | 7.8 | 3.07 | 1.04 | 2.72 | 0.65 | 0.25 | 0.00 | 0.72 | 0.97 |
| **FeLV-09** | 4 | 38 | 12.2 | 8.55 | 6.10 | 3.78 | 1.83 | 94 | 2.0 | 15 | 9.4 | 3.11 | 1.33 | 3.73 | 0.49 | 0.06 | 0.00 | 1.09 | 0.79 |
| **FeLV-09** | 10 | 38 | 12.2 | 8.55 | 7.10 | 4.15 | 2.85 | 94 | 2.0 | 15 | 9.4 | 3.11 | 1.33 | 3.73 | 0.49 | 0.06 | 0.00 |  | 0.73 |
| **FeLV-10** | 0 | 10 | 3.4 | 2.04 | 0.01 | 1.11 | 10.95 | 51 | 1.0 | 22 | 6.0 | 3.48 | 0.95 | 1.06 | 1.38 | 0.84 | 11.45 | 0.64 | 0.55 |
| **FeLV-10** | 2 | 17 | 5.80 | 3.54 | 3.00 | 1.20 | 1.31 | 52 | 1.3 | 25 | 9.6 | 3.42 | 1.13 | 4.34 | 0.55 | 1.27 | 10.52 |  | 0.69 |
| **FeLV-11** | 0 | 34 | 10.6 | 7.17 | 1.80 | 0.05 | 1.66 | 58 | 1.1 | 24 | 6.5 | 4.05 | 0.98 | 0.94 | 1.71 | 0.67 | 0.98 | 0.98 | 0.77 |
| **FeLV-11** | 2 | 40 | 12.5 | 7.63 | 24.10 | 17.35 | 5.06 | 53 | 1.1 | 20 | 9.4 | 3.34 | 1.08 | 4.10 | 0.55 | 0.68 | 0.00 | 1.53 | 0.87 |
| **FeLV-11** | 4 | 34 | 12.4 | 14.10 | 8.50 | 4.93 | 3.06 |  |  |  |  |  |  |  |  | 0.67 | 0.90 | 2.21 | 0.92 |
| **FeLV-12** | 0 | 42 | 13.4 | 10.03 | 10.80 | 1.62 | 7.88 | 66 | 1.2 | 31 | 6.6 | 3.46 | 1.44 | 1.05 | 1.10 | 0.05 | 0.21 |  | 1.07 |
| **FeLV-12** | 2 | 36 | 11.6 | 9.03 | 24.60 | 16.24 | 6.64 | 74 | 0.8 | 44 | 8.0 | 2.83 | 1.16 | 3.25 | 0.55 | 0.30 | 0.19 | 1.56 | 0.77 |
| **FeLV-12** | 4 | 32 | 11.8 | 11.31 | 20.40 | 15.49 | 4.35 | 58 | 1.2 | 35 | 10.4 | 2.54 | 1.28 | 6.32 | 0.34 | 0.33 | 0.46 |  | 0.82 |
| **FeLV-13** | 0 | 35 | 11.4 | 7.27 | 7.30 | 4.16 | 2.70 | 57 | 1.5 | 19 | 6.6 | 4.08 | 0.93 | 1.05 | 1.62 | 0.58 | 0.00 | 3.91 | 0.79 |
| **FeLV-13** | 2 | 41 | 12.5 | 8.49 | 12.50 | 8.88 | 2.88 | 54 | 1.1 | 36 | 8.4 | 3.41 | 1.14 | 2.92 | 0.68 | 0.25 | 0.00 | 2.25 | 0.93 |
| **FeLV-13** | 4 | 37 | 12.1 | 8.26 | 7.00 | 1.40 | 4.13 | 110 | 1.9 | 20 | 10.2 | 3.56 | 1.44 | 3.60 | 0.54 | 0.06 | 0.00 | 2.39 | 0.99 |
| **FeLV-14** | 0 | 30 | 10.6 | 11.50 | 5.30 | 2.81 | 1.91 | 47 | 1.2 | 37 | 6.2 | 2.52 | 0.98 | 2.25 | 0.68 | 0.24 | 0.00 | 2.45 | 0.63 |
| **FeLV-14** | 2 | 35 | 13.7 |  | 7.90 | 1.26 | 6.48 | 52 | 1.7 | 36 | 7.6 | 4.62 | 0.96 | 1.14 | 1.55 | 0.62 | 0.00 |  | 0.49 |
| **FeLV-14** | 4 | 38 | 12.5 | 8.02 | 7.20 | 3.89 | 2.45 | 62 | 1.3 | 28 | 6.4 | 4.20 | 1.07 | 0.71 | 1.91 | 1.51 | 0.00 | 3.16 | 0.77 |
| **FeLV-14** | 10 | 26 | 6.8 |  | 19.80 | 15.05 | 4.55 | 49 | 1.1 | 15 | 6.8 | 3.30 | 1.13 | 1.58 | 0.94 | 0.65 | 0.00 | 3.28 | 0.84 |
| **FeLV-15** | 0 | 17 | 4.8 | 4.15 | 6.50 | 1.30 | 5.20 | 47 | 0.8 | 39 | 5.8 | 2.75 | 1.06 | 1.35 | 0.90 | 0.50 | 3.46 | 0.66 | 0.60 |
| **FeLV-15** | 2 | 26 | 7.8 | 7.63 | 12.90 | 7.61 | 3.61 | 47 | 0.8 | 25 | 8.2 | 3.06 | 1.13 | 3.16 | 0.60 | 0.17 | 0.00 | 1.12 | 0.88 |
| **FeLV-15** | 4 | 28 | 7.50 | 7.74 | 8.50 | 4.93 | 3.06 |  |  |  | 8.6 | 3.21 | 0.80 | 3.45 | 0.59 | 0.46 | 3.29 | 1.32 | 0.59 |
| **FeLV-16** | 0 | 47 | 14.3 | 10.36 | 4.10 | 2.17 | 1.35 | 56 | 1.0 | 20 | 6.2 | 3.53 | 0.85 | 1.07 | 1.33 | 0.20 | 0.00 | 2.12 | 0.87 |
| **FeLV-16** | 2 | 29 | 9.3 | 5.41 | 21.00 | 14.28 | 6.51 |  |  |  | 7.0 | 3.29 | 1.38 | 1.55 | 0.89 | 0.75 | 0.00 | 3.16 | 0.61 |
| **FeLV-16** | 4 | 30 | 8.9 | 7.01 | 7.70 | 2.31 | 4.39 | 57 | 0.8 | 24 | 6.2 | 2.60 | 1.13 | 1.92 | 0.72 | 0.94 | 0.00 | 2.68 | 1.25 |
| **FeLV-17** | 0 | 43 | 13.7 | 10.53 | 8.40 | 6.22 | 1.76 | 47 | 1.4 | 17 | 6.6 | 3.79 | 0.97 | 1.12 | 1.35 | 0.37 | 0.00 | 2.57 | 0.86 |
| **FeLV-17** | 2 | 29 | 9.1 | 5.41 | 5.40 | 2.43 | 2.59 | 44 | 1.1 |  | 6.6 | 3.52 | 1.23 | 1.26 | 1.15 | 0.21 | 0.00 | 2.6 | 0.87 |
| **FeLV-17** | 4 |  |  |  |  |  |  | 84 | 1.0 |  | 7.8 | 3.35 | 1.32 | 2.16 | 0.75 | 1.31 | 0.00 | 2.72 | 1.21 |
| **FeLV-17** | 10 | 30 | 8.70 | 7.67 | 8.70 | 4.76 | 3.16 | 55 | 1.3 | 42 | 8.4 | 3.85 | 1.15 | 2.03 | 0.85 | 0.66 | 0.00 |  | 0.49 |
| **FeLV-18** | 0 | 36 | 12.5 | 9.33 | 9.60 | 1.15 | 7.68 | 74 | 1.8 | 25 | 6.4 | 4.22 | 0.74 | 0.86 | 1.94 | 0.05 | 0.00 | 1.52 | 0.62 |
| **FeLV-18** | 2 | 36 | 10.7 | 9.27 | 22.40 | 13.66 | 5.60 | 60 | 0.9 | 85 | 7.6 | 3.33 | 1.11 | 2.43 | 0.78 | 0.32 | 0.00 | 1.66 | 0.81 |
| **FeLV-18** | 10 | 20 | 6.5 | 4.25 | 10.70 | 6.85 | 2.57 | 31 | 0.8 | 15 | 7.0 | 3.17 | 0.94 | 2.09 | 0.83 | 0.06 | 0.00 | 1.54 | 0.72 |
| **FeLV-19** | 0 | 36 | 11.9 | 7.08 | 12.50 | 9.75 | 2.25 | 58 | 1.3 | 32 | 8.2 | 4.05 | 1.16 | 2.04 | 0.98 | 0.33 | 0.10 |  | 0.64 |
| **FeLV-19** | 2 | 44 | 13.5 |  | 6.60 | 2.38 | 4.09 | 61 | 1.7 | 26 | 9.4 | 3.70 | 1.14 | 3.85 | 0.65 | 0.08 | 0.00 | 0.87 | 0.94 |
| **FeLV-19** | 4 | 47 | 15 | 11.60 | 8.50 | 4.93 | 3.06 | 52 | 1.3 | 25 | 9.6 | 3.42 | 1.13 | 4.34 | 0.55 | 0.11 | 0.00 | 0.84 | 0.96 |
| **FeLV-20** | 0 | 22 | 7.50 | 4.45 | 0.01 | 6.70 | 3.87 | 54 | 1.3 | 30 | 7.2 | 4.01 | 1.34 | 1.19 | 1.26 | 0.40 | 0.15 | 1.98 | 0.65 |
| **FeLV-20** | 2 | 52 | 16.6 | 11.99 | 8.40 | 3.95 | 3.53 | 66 | 1.1 | 35 | 8.2 | 2.63 | 1.53 | 2.11 | 0.47 | 0.06 | 0.00 | 2.2 | 0.87 |
| **FeLV-20** | 4 | 37 | 11.1 | 7.11 | 5.90 | 4.07 | 0.83 | 45 | 2.0 | 22 | 8.8 | 2.94 | 1.36 | 3.55 | 0.50 | 0.06 | 0.15 | 1.05 | 0.80 |
| **FeLV-20** | 10 | 21 | 6.09 | 4.86 | 58.40 | 57.23 | 1.17 | 35 | 0.5 | 27 | 6.4 | 3.25 | 1.16 | 1.16 | 1.03 | 0.26 | 0.77 | 2.34 | 0.65 |
| **FeLV-21** | 0 | 24 | 10.2 | 8.54 | 0.01 | 7.33 | 3.05 | 52 | 0.9 | 27 | 6.7 | 4.31 | 0.78 | 1.08 | 1.80 | 0.88 | 2.05 | 2.08 | 0.88 |
| **FeLV-21** | 2 | 28 | 8.4 | 7.52 | 9.80 | 6.47 | 1.08 | 52 | 0.7 | 25 | 7.6 | 2.90 | 1.39 | 2.71 | 0.62 | 0.62 | 0.18 | 1.72 | 0.86 |
| **FeLV-22** | 0 | 39 | 13.2 | 10.12 | 6.30 | 3.72 | 2.33 | 52 | 1.6 | 18 | 6.4 | 3.59 | 0.89 | 1.23 | 1.27 | 0.61 | 0.00 | 1.87 | 0.76 |
| **FeLV-22** | 2 | 38 | 10.2 | 8.61 | 18.90 | 12.70 | 4.20 | 56 | 1.3 | 19 | 6.6 |  |  |  |  | 0.40 | 0.00 | 2.31 | 0.67 |
| **FeLV-22** | 4 | 39 | 12.3 | 7.93 | 7.40 | 3.70 | 3.18 | 35 | 1.0 | 44 | 6.8 | 4.52 | 1.05 | 0.54 | 1.98 | 0.58 | 0.00 |  | 1.02 |
| **FeLV-22** | 10 | 34 | 12.2 | 7.23 | 5.00 | 2.20 | 2.65 | 52 | 1.5 | 54 | 8.4 | 4.11 | 1.16 | 2.21 | 0.96 | 1.03 | 0.78 | 2.31 | 0.39 |
| **FeLV-23** | 0 | 41 | 13.4 | 9.08 | 4.60 | 2.12 | 2.16 | 57 | 1.7 | 54 | 6.8 | 3.51 | 1.24 | 1.52 | 1.07 | 0.53 | 1.87 | 2.23 | 0.52 |
| **FeLV-23** | 2 | 29 | 8.6 | 6.48 | 4.20 | 1.97 | 1.64 | 47 | 1.7 | 13 | 8.6 | 2.74 | 1.06 | 4.15 | 0.47 | 0.48 | 0.00 | 2.39 | 0.62 |
| **FeLV-23** | 4 | 38 | 12.7 | 9.00 | 5.40 | 2.05 | 2.92 |  |  |  | 6.2 |  |  |  |  | 0.04 | 0.00 | 3.59 | 0.87 |
| **FeLV-23** | 10 | 29 | 10.7 | 7.64 | 5.40 | 2.97 | 2.00 | 59 | 1.0 | 33 | 8.2 |  |  |  |  | 1.06 | 0.03 | 3.1 | 0.72 |
| **FeLV-24** | 0 | 18 | 6.2 | 4.83 | 14.20 | 11.08 | 2.84 | 58 | 1.5 | 45 | 7.4 | 3.61 | 1.16 | 1.93 | 0.95 | 0.10 | 0.00 | 2.7 | 0.72 |
| **FeLV-24** | 2 | 23 | 6.8 | 5.84 | 29.40 | 24.11 | 3.53 | 70 | 1.1 | 37 | 8.2 | 2.85 | 1.45 | 2.92 | 0.53 | 0.05 | 0.00 |  | 0.64 |
| **FeLV-24** | 4 | 32 | 10.1 | 9.17 | 7.10 | 2.20 | 3.34 | 57 | 0.9 | 24 | 7.4 | 2.97 | 1.22 | 2.49 | 0.67 | 0.65 | 0.68 | 2.34 | 1.09 |
| **FeLV-24** | 10 | 54 | 16.2 | 8.33 | 19.50 | 15.30 | 2.80 | 62 | 1.2 | 15 | 9.6 | 3.01 | 1.23 | 4.73 | 0.46 | 1.24 | 0.00 | 3.11 | 0.71 |
| **FeLV-25** | 0 | 31 | 10.1 | 6.64 | 7.17 | 3.60 | 3.46 | 49 | 0.9 | 47 | 5.8 | 2.92 | 1.03 | 1.23 | 1.01 | 0.43 | 0.81 | 0.79 | 0.79 |
| **FeLV-25** | 4 | 36 | 14.2 |  | 9.43 | 2.76 | 6.48 | 54 | 1.6 | 39 | 7.4 | 4.52 | 0.98 | 1.05 | 1.48 | 0.05 | 0.00 | 1.67 | 1.05 |
| **FeLV-26** | 0 | 50 | 15.4 | 10.54 | 8.70 | 2.10 | 4.40 | 72 | 1.3 | 32 | 8.2 | 4.05 | 1.25 | 1.83 | 0.98 | 0.54 | 0.89 |  | 0.64 |
| **FeLV-26** | 10 | 25 | 7.90 |  | 4.40 | 2.90 | 1.45 | 48 | 0.9 | 29 | 7.0 | 3.68 | 1.24 | 1.39 | 1.11 | 0.79 | 0.97 | 1.24 | 0.86 |
| **FeLV-27** | 0 | 20 | 7.1 | 5.60 | 10.30 | 7.11 | 2.78 | 37 | 0.6 | 24 | 10.5 | 2.56 | 1.53 | 6.25 | 0.30 | 0.10 | 0.00 | 1.6 | 0.84 |
| **FeLV-27** | 2 | 45 | 14 | 10.44 | 13.20 | 8.05 | 4.36 | 72 | 1.5 | 36 | 8.4 | 3.30 | 1.41 | 2.69 | 0.65 | 0.69 | 0.96 | 1.51 | 0.66 |
| **FeLV-27** | 4 | 38 | 11.7 | 7.76 | 11.30 | 5.65 | 4.75 | 46 | 1.2 | 20 | 8.2 | 2.44 | 1.51 | 3.19 | 0.42 | 1.11 | 0.99 | 2.29 | 0.61 |
| **FeLV-27** | 10 | 25 | 7.9 | 5.85 | 7.90 | 4.27 | 3.63 | 20 | 0.6 | 6 | 6.6 | 2.61 | 1.23 | 1.96 | 0.65 | 0.81 | 2.77 | 2.82 | 0.74 |
