# Supplementary material for: Clinical and Hematological Follow-Up of Long-Term Oral Therapy with Type-I Interferon in Cats Naturally Infected with Feline Leukemia Virus or Feline Immunodeficiency Virus
Source: Animals (Basel). 2020 Aug 20;10(9):1464. doi: 10.3390/ani10091464 (PMC7552327; doi:10.3390/ani10091464)
Supplement: Supplementary file 1 [file animals-10-01464-s001.zip › Table S1 v2.docx]

**Table S1**. Follow-up of the clinical score (CS) of each individual cat infected either by FeLV (upper three tables) or by FIV (bottom three tables) belonging to each clinical group (CG1 , GG2 and CG3) at each moment of treatment with rHu-IFN. CG, clinical group. M0, beginning of treatment. M2, two months (±15 days) after beginning of treatment (mid-treatment). M4, four months (±15 days) after beginning of treatment (end of treatment). M10, 10 months (±2 months) after beginning of treatment. D, death. Blank cells indicate that the cat was not brought to the clinic at that time point. The rating of the clinical score has been published previously (Collado et al., 2012).

| **CG1** | FeLV-03 | FeLV-07 | FeLV-09 | FeLV-13 | FeLV-16 | FeLV-17 | FeLV-18 | FeLV-21 | FeLV-22 | FeLV-26 | FeLV-27 |
| --- | --- | --- | --- | --- | --- | --- | --- | --- | --- | --- | --- |
| M0 | 0 | 0 | 0 | 0 | 0 | 0 | 0 | 0 | 0 | 0 | 0 |
| M2 | 0 | 0 | 0 | 0 | 0 | 0 | 0 | 0 | 0 |  | 0 |
| M4 | 0 | 0 | 0 | 0 | 0 | 0 |  |  | 0 |  | 0 |
| M10 |  | 0 | 0 |  |  | 0 | 0 |  | 0 | 0 | 0 |
|  |  |  |  |  |  |  |  |  |  |  |  |
| **CG2** | FeLV-01 | FeLV-02 | FeLV-04 | FeLV-05 | FeLV-08 | FeLV-14 | FeLV-15 | FeLV-19 | FeLV-24 |  |  |
| M0 | 5 | 4 | 4 | 4 | 4 | 4 | 5 | 2 | 1 |  |  |
| M2 | 5 | 7 | 3 | 4 | 4 | 3 | 5 | 0 | 0 |  |  |
| M4 | 3 | 5 | 3 | 2 | 2 | 2 | 0 | 0 | 0 |  |  |
| M10 | 1 |  |  |  |  | 2 | 0 |  |  |  |  |
|  |  |  |  |  |  |  |  |  |  |  |  |
| **CG3** | FeLV-06 | FeLV-10 | FeLV-11 | FeLV-12 | FeLV-20 | FeLV-23 | FeLV-25 |  |  |  |  |
| M0 | 8 | 8 | 7 | 7 | 13 | 6 | 7 |  |  |  |  |
| M2 | 8 | 2 | 7 | 5 | 13 | 2 | 7 |  |  |  |  |
| M4 | D | 2 | D | 4 | 8 | 0 | 0 |  |  |  |  |
| M10 | D |  | D |  |  | 0 | 0 |  |  |  |  |

| **CG1** | FIV-01 | FIV-05 | FIV-11 | FIV-12 | FIV-18 | FIV-23 | FIV-25 | FIV-28 | FIV-31 |  |  |  |  |
| --- | --- | --- | --- | --- | --- | --- | --- | --- | --- | --- | --- | --- | --- |
| M0 | 0 | 0 | 0 | 0 | 0 | 0 | 0 | 0 | 0 |  |  |  |  |
| M2 | 0 | 0 | 0 |  |  | 0 |  | 0 | 0 |  |  |  |  |
| M4 | 0 |  | 0 | 0 | 0 |  | 0 | 0 | 0 |  |  |  |  |
| M10 | 0 | 0 | 0 | 0 | 0 | 0 | 0 | 0 |  |  |  |  |  |
|  |  |  |  |  |  |  |  |  |  |  |  |  |  |
| **CG2** | FIV-02 | FIV-03 | FIV-04 | FIV-06 | FIV-09 | FIV-10 | FIV-13 | FIV-16 | FIV-17 | FIV-22 | FIV-27 | FIV-29 | FIV-30 |
| M0 | 4 | 3 | 3 | 5 | 2 | 4 | 5 | 2 | 4 | 3 | 4 | 2 | 2 |
| M2 | 0 | 0 | 1 | 0 |  | 2 | 1 | 2 | 2 | 4 | 3 | 0 |  |
| M4 | 0 |  | 0 | 0 | 0 | 0 |  |  | 0 | 2 | 2 |  | 0 |
| M10 | 0 | 0 |  | 0 | 0 | 0 | 0 |  | 0 | 8 | 0 | 0 | 2 |
|  |  |  |  |  |  |  |  |  |  |  |  |  |  |
| **CG3** | FIV-07 | FIV-08 | FIV-14 | FIV-15 | FIV-19 | FIV-20 | FIV-21 | FIV-24 | FIV-26 |  |  |  |  |
| M0 | 6 | 7 | 9 | 7 | 6 | 6 | 6 | 6 | 7 |  |  |  |  |
| M2 | 3 | 2 |  | 5 | 5 | 1 | 2 | 6 | 3 |  |  |  |  |
| M4 | 3 | 2 | 4 | 4 | 0 | 1 | 2 | 0 | 0 |  |  |  |  |
| M10 | 1 | 2 | D |  | 0 | 0 |  | 0 |  |  |  |  |  |
