# Supplementary material for: Clinical and Hematological Follow-Up of Long-Term Oral Therapy with Type-I Interferon in Cats Naturally Infected with Feline Leukemia Virus or Feline Immunodeficiency Virus
Source: Animals (Basel). 2020 Aug 20;10(9):1464. doi: 10.3390/ani10091464 (PMC7552327; doi:10.3390/ani10091464)
Supplement: Supplementary file 1 [file animals-10-01464-s001.zip › Table S3 v2.docx]

| Table S2. Individual results of the FIV-infected cats analyzed in the study. ID, identification of each cat. M, month of the visit as follows: M0, beginning of treatment. M2, two months (±15 days) after beginning of treatment (mid-treatment). M4, four months (±15 days) after beginning of treatment (end of treatment). M10, 10 months (±2 months) after beginning of treatment. PCV, packed cell volume (reference range 26-45%). Hgb, hemoglobin concentration (reference range 8-15 g/dL). RBC, red blood cell count (reference range 5-10 x 10^6^ cells/µL). Leuk, leukocyte count (reference range 5.5-19.5 x 10^3^ cells/µL). Ntr, segmented neutrophil count (reference range 2.5-12.5 x10^3^ cells/µL). Lym, lymphocyte count (reference range 1.5-7.7 x10^3^ cells/µL). Urea (reference range 38-71 mg/dL). Creat, creatinine (reference range 0.7-1.8 mg/dL). ALT, alanine amino transferase (reference range 30-100 UI/L). Prot, total proteins (reference range 5.7-7.9 g/dL). Alb, albumin (reference range 2.3-4.0 g/dL). α-s, serum α-globulins (reference range 0.5-1.5 g/dL). γ-s, γ-globulins (reference range 0.5-1.5 g/dL). A/G, albumin/globulins ratio (reference range 0.7-1.3). CD4/CD8, CD4:CD8 ratio. rtPCR, real time PCR. Blank cells indicate that the analysis could not be done at that time point.**id** | **M** | **PCV** | **Hgb** | **RBC x 10^6^** | **Leuk x 10^3^** | **Ntr x 10^3^** | **Lym x 10^3^** | **Urea** | **Creat** | **ALT** | **Prot** | **Alb** | **α-s** | **γ-s** | **A/G** | **CD4**  **CD8** | **rt**  **PCR** |
| --- | --- | --- | --- | --- | --- | --- | --- | --- | --- | --- | --- | --- | --- | --- | --- | --- | --- |
| **FIV-01** | 0 | 38 | 11.6 | 8.24 | 4.80 | 1.80 | 2.40 | 56 | 1.1 | 44 | 7.7 | 3.45 | 0.85 | 2.37 | 0.81 | 0.75 | 0.89 |
| **FIV-01** | 2 | 47 | 14.7 | 10.63 | 4.30 | 1.94 | 2.11 | 48 | 0.9 | 15 | 8.6 | 1.77 | 1.24 | 4.73 | 0.26 | 1.12 | 0.90 |
| **FIV-01** | 4 | 40 | 11.7 | 8.21 | 7.00 | 3.78 | 2.10 | 51 | 0.7 | 15 | 8.4 | 2.98 | 1.19 | 3.57 | 0.55 | 1.68 | 0.77 |
| **FIV-01** | 10 | 41 | 12.4 | 8.95 | 7.70 | 3.39 | 3.85 | 64 | 1.4 | 15 | 8.0 | 3.56 | 1.29 | 2.49 | 0.80 | 1.35 | 0.97 |
| **FIV-02** | 0 | 38 | 14.5 | 7.32 | 9.10 | 3.30 | 4.20 | 55 | 1.3 | 42 | 8.4 | 3.85 | 1.15 | 2.60 | 0.84 |  | 0.94 |
| **FIV-02** | 2 | 32 | 9.4 | 6.33 | 7.30 | 4.96 | 1.46 | 154 | 1.9 | 50 | 8.0 | 3.31 | 1.12 | 2.77 | 0.71 | 1.98 | 0.94 |
| **FIV-02** | 4 | 33 | 10.7 | 8.01 | 9.90 | 5.15 | 3.56 |  | 1.1 | 15 | 7.6 | 2.83 | 1.34 | 2.90 | 0.59 | 2.4 | 0.87 |
| **FIV-02** | 10 | 41 | 12.2 | 9.05 | 8.90 | 3.74 | 4.36 | 53 | 1.6 | 18 | 8.6 | 4.57 | 1.17 | 2.28 | 1.13 | 2.35 | 0.89 |
| **FIV-03** | 0 | 37 | 12.9 | 7.22 | 7.20 | 4.61 | 2.02 | 63 | 1.3 | 22 | 9.2 | 4.04 | 1.69 | 2.74 | 0.78 | 0.54 | 0.79 |
| **FIV-03** | 4 | 33 | 9.3 | 7.31 | 18.40 | 10.12 | 8.10 | 45 | 0.6 | 11 | 8.8 | 2.96 | 1.67 | 3.40 | 0.51 | 0.87 | 0.88 |
| **FIV-03** | 10 | 44 | 13.3 | 10.18 | 10.00 | 5.00 | 3.80 | 54 | 1.1 | 48 | 7.2 | 3.31 | 1.03 | 1.83 | 0.85 |  | 0.67 |
| **FIV-04** | 0 | 44 | 12.9 | 7.26 | 15.30 | 5.50 | 6.80 | 74 | 1.5 | 31 | 7.6 | 3.89 | 1.06 | 1.82 | 1.05 | 0.72 | 0.74 |
| **FIV-04** | 2 | 19 | 7 | 4.79 | 3.40 | 2.01 | 1.22 | 60 | 1.2 | 10 | 7.4 | 3.25 | 0.88 | 2.37 | 0.79 | 1.24 | 0.91 |
| **FIV-04** | 4 | 37 | 13.2 | 10.43 | 4.20 | 1.68 | 1.72 | 54 | 1.1 | 31 | 7.0 | 3.13 | 0.97 | 2.07 | 0.81 | 1.15 | 0.80 |
| **FIV-05** | 0 | 34 | 10.1 | 5.68 | 4.60 | 1.84 | 2.53 | 48 | 1.5 | 25 | 8.4 | 4.07 | 1.07 | 2.40 | 0.94 | 1.14 | 0.88 |
| **FIV-05** | 2 | 35 | 11.5 | 7.20 | 8.50 | 2.98 | 5.51 | 51 | 1.4 | 24 | 8.9 | 3.50 | 1.14 | 4.05 | 0.69 | 1.86 | 0.98 |
| **FIV-05** | 10 | 45 | 10.5 | 8.85 | 9.90 | 3.10 | 4.60 | 48 | 1.2 | 18 | 7.6 | 2.94 | 0.97 | 3.15 | 0.57 | 1.1 | 0.79 |
| **FIV-06** | 0 | 30 | 9.2 | 6.35 | 25.90 | 19.68 | 4.14 | 51 | 1.2 | 13 | 7.8 | 3.42 | 1.30 | 1.83 | 0.78 | 0.27 | 0.90 |
| **FIV-06** | 2 | 41 | 10.8 |  | 19.60 | 11.96 | 5.49 | 56 | 1.1 | 46 | 10.0 | 3.55 | 1.75 | 3.78 | 0.55 | 1.94 | 1.03 |
| **FIV-06** | 4 | 21 | 7.1 | 4.37 | 6.20 | 2.91 | 2.60 | 70 | 0.9 | 23 | 8.2 | 4.87 | 0.95 | 1.64 | 1.47 |  | 0.94 |
| **FIV-06** | 10 | 34 | 10.9 | 8.44 | 8.20 | 4.76 | 2.79 | 50 | 1.3 | 24 | 7.6 | 3.39 | 0.97 | 2.30 | 0.81 | 0.76 | 0.71 |
| **FIV-07** | 0 | 31 | 11.3 | 8.65 | 13.40 | 9.78 | 3.35 | 47 | 0.9 | 38 | 7.4 | 3.67 | 1.13 | 1.95 | 0.98 | 0.64 | 0.88 |
| **FIV-07** | 2 | 32 | 11.8 |  | 25.50 | 15.30 | 7.40 | 48 | 1.1 | 15 | 8.6 | 2.49 | 1.49 | 3.95 | 0.41 | 0.9 | 0.90 |
| **FIV-07** | 4 |  |  |  |  |  |  | 47 | 1.2 | 13 | 8.4 | 2.89 | 0.97 | 3.75 | 0.53 | 0.77 | 0.90 |
| **FIV-07** | 10 | 43 | 10.7 | 9.46 | 8.00 | 1.40 | 4.90 | 59 | 1.3 | 22 | 8.2 | 3.46 | 1.15 | 2.52 | 0.73 | 0.68 | 0.87 |
| **FIV-08** | 0 | 26 | 6.8 | 7.24 | 19.80 | 15.05 | 4.55 | 49 | 1.1 | 15 | 6.8 | 3.30 | 1.13 | 1.58 | 0.94 |  | 0.88 |
| **FIV-08** | 2 | 36 | 11.6 | 8.20 | 7.10 | 4.76 | 1.85 | 66 | 1.4 | 22 | 6.8 | 3.46 | 1.19 | 1.60 | 1.04 | 0.65 | 0.95 |
| **FIV-08** | 4 | 32 | 9.7 | 6.92 | 10.60 | 6.25 | 3.50 | 58 | 0.8 | 21 | 8.0 | 2.91 | 0.99 | 3.42 | 0.57 | 1.67 | 0.91 |
| **FIV-08** | 10 | 35 | 8.7 | 6.50 | 6.20 | 1.00 | 4.10 | 57 | 1.3 | 22 | 7.0 | 3.74 | 1.02 | 1.42 | 1.15 | 0.59 | 0.79 |
| **FIV-09** | 0 | 19 | 5.9 | 3.85 | 4.30 | 0.99 | 2.58 | 82 | 1.5 | 37 | 9.0 | 3.35 | 1.12 | 4.15 | 0.60 | 1.35 | 0.77 |
| **FIV-09** | 4 | 33 | 10.1 | 6.34 | 17.90 | 5.19 | 11.28 | 50 | 1.5 | 21 | 8.6 | 2.84 | 1.35 | 3.79 | 0.49 | 1.76 | 0.89 |
| **FIV-09** | 10 | 42 | 12.9 | 9.48 | 10.80 | 2.59 | 7.78 | 47 | 1.3 | 29 | 7.8 | 3.71 | 0.90 | 2.21 | 0.90 | 2.54 | 0.98 |
| **FIV-10** | 0 | 28 | 9.4 | 6.87 | 8.20 | 3.69 | 4.26 | 43 | 1.3 | 12 | 6.0 | 3.25 | 0.80 | 1.27 | 1.18 | 0.82 | 0.74 |
| **FIV-10** | 2 | 34 | 11.2 | 7.99 | 8.00 | 2.72 | 3.68 | 42 | 1.5 | 20 | 8.2 | 2.55 | 0.86 | 4.06 | 0.45 | 1.12 | 0.94 |
| **FIV-10** | 4 |  |  |  |  |  |  | 45 | 0.9 | 14 | 7.8 | 2.92 | 1.02 | 3.06 | 0.60 |  | 0.97 |
| **FIV-10** | 10 | 38 | 11.8 | 8.17 | 9.00 | 3.06 | 5.76 | 69 | 1.4 | 28 | 6.6 | 3.63 | 1.09 | 1.24 | 1.22 |  | 0.80 |
| **FIV-11** | 0 | 25 | 9.6 | 6.77 | 6.70 | 1.61 | 4.49 | 42 | 1.1 | 39 | 8.0 | 3.62 | 1.07 | 2.69 | 0.83 | 0.48 | 0.68 |
| **FIV-11** | 2 | 30 | 10.6 | 8.20 | 13.50 | 4.59 | 7.43 | 40 | 0.8 | 14 | 7.2 | 3.61 | 1.10 | 1.81 | 1.01 | 0.89 | 0.76 |
| **FIV-11** | 4 | 33 | 10.7 | 6.83 | 16.10 | 6.44 | 8.21 | 57 |  | 27 | 7.6 | 3.22 | 0.98 | 2.79 | 0.74 | 1.3 | 0.81 |
| **FIV-11** | 10 | 29 | 8.2 | 6.82 | 18.20 | 3.40 | 10.70 | 169 | 6.0 | 12 | 8.2 | 2.20 | 1.14 | 4.05 | 0.37 | 1.04 | 0.93 |
| **FIV-12** | 0 | 40 | 12.3 | 8.47 | 15.30 | 3.00 | 1.70 | 39 | 1.3 | 27 | 6.2 | 2.11 | 0.88 | 1.47 | 0.52 |  | 0.75 |
| **FIV-12** | 4 |  |  |  |  |  |  | 28 | 1.3 | 49 | 6.6 |  |  |  |  |  | 0.74 |
| **FIV-12** | 10 | 38 | 12 | 6.76 | 5.20 | 1.82 | 2.91 | 65 | 1.2 | 44 | 7.6 | 2.84 | 1.05 | 2.96 | 0.60 | 2.48 | 0.94 |
| **FIV-13** | 0 | 18 | 5.6 | 3.67 | 18.70 | 16.46 | 0.56 | 110 | 1.5 | 135 | 7.8 | 3.03 | 1.39 | 2.59 | 0.64 | 0.86 | 0.68 |
| **FIV-13** | 2 | 32 | 8.8 | 7.08 | 4.00 | 0.20 | 3.68 | 45 | 1.3 | 75 | 6.8 | 2.79 | 0.87 | 2.22 | 0.70 | 1.09 | 0.82 |
| **FIV-13** | 10 | 42 | 13.5 | 8.24 | 5.60 | 3.80 | 2.00 | 62 | 1.4 | 18 | 7.6 | 4.38 | 1.08 | 1.32 | 1.36 |  | 0.72 |
| **FIV-14** | 0 | 37 | 12 | 7.06 | 5.90 | 2.30 | 2.71 | 56 | 1.5 | 37 | 8.6 | 3.36 | 1.47 | 2.87 | 0.64 | 0.42 | 0.80 |
| **FIV-14** | 4 | 37 | 11.3 | 9.20 | 17.10 | 13.68 | 2.91 | 51 | 1.2 | 24 | 8.0 |  |  |  |  | 0.88 | 0.79 |
| **FIV-15** | 0 | 22 | 5.4 | 4.54 | 6.20 | 2.34 | 3.75 | 87 | 2.0 | 41 | 8.2 | 3.24 | 1.24 | 3.34 | 0.66 | 0.74 | 0.87 |
| **FIV-15** | 2 | 35 | 13 | 9.38 | 8.70 | 2.52 | 5.66 | 47 | 0.8 | 8 | 11.0 | 3.16 | 1.69 | 5.04 | 0.40 | 1.05 | 0.98 |
| **FIV-15** | 4 | 45 | 13 | 9.15 | 9.00 | 6.80 | 1.30 |  |  |  |  |  |  |  |  | 0.58 | 0.80 |
| **FIV-16** | 0 | 37 | 11.5 | 7.62 | 9.40 | 6.30 | 1.10 | 48 | 1.2 | 26 | 6.4 | 3.37 | 0.87 | 1.30 | 1.11 | 0.34 | 0.84 |
| **FIV-16** | 2 | 39 | 12.1 | 8.73 | 6.80 | 3.40 | 2.58 | 52 | 1.3 | 14 | 7.5 | 3.71 | 1.02 | 2.02 | 0.98 | 0.75 | 1.03 |
| **FIV-17** | 0 | 38 | 12.5 | 8.02 | 7.20 | 3.89 | 2.45 | 62 | 1.3 | 28 | 9.0 | 3.53 | 1.63 | 3.22 | 0.65 | 1.18 | 0.81 |
| **FIV-17** | 2 | 42 |  |  | 7.00 | 3.01 | 3.71 | 53 | 1.0 | 9 | 6.8 | 3.31 | 1.13 | 1.75 | 0.95 |  | 0.74 |
| **FIV-17** | 4 | 44 | 12.3 | 8.54 | 9.80 | 3.15 | 3.48 | 55 | 1.1 | 10 | 10.9 | 3.51 | 1.09 | 5.68 | 0.50 | 1.56 | 0.69 |
| **FIV-17** | 10 | 43 | 13 | 9.82 | 5.60 | 0.80 | 4.20 | 53 | 1.3 | 31 | 7.4 |  |  |  |  | 1.42 | 0.82 |
| **FIV-18** | 0 | 21 | 6.09 | 4.86 | 58.40 | 57.23 | 1.17 | 35 | 0.5 | 27 | 6.4 | 3.25 | 1.16 | 1.16 | 1.03 | 0.79 | 0.79 |
| **FIV-18** | 4 | 31 | 9.6 | 6.09 | 17.80 | 11.75 | 3.20 | 67 | 1.9 | 16 | 9.6 | 2.57 | 0.88 | 5.45 | 0.37 | 0.96 | 0.87 |
| **FIV-18** | 10 | 31 | 9.1 | 6.99 | 31.00 | 26.97 | 3.41 | 42 | 1.1 | 81 | 5.0 | 1.69 | 0.66 | 2.60 | 0.51 | 0.72 | 0.94 |
| **FIV-19** | 0 | 25 | 7.9 | 6.02 | 4.40 | 2.90 | 1.45 | 48 | 0.9 | 29 | 7.0 | 3.68 | 1.24 | 1.39 | 1.11 | 1.14 | 0.88 |
| **FIV-19** | 2 | 23 | 6.9 | 5.49 | 15.50 | 9.15 | 4.50 | 46 | 1.3 | 18 | 7.0 | 3.23 | 0.84 | 2.31 | 0.86 | 1.58 | 0.87 |
| **FIV-19** | 4 | 42 | 11.8 | 8.25 | 9.10 | 3.20 | 3.80 | 57 | 1.0 | 9 | 8.8 | 2.83 | 1.09 | 4.20 | 0.47 | 1.32 | 0.75 |
| **FIV-19** | 10 | 39 | 9.9 | 5.51 | 8.30 | 3.50 | 1.70 | 48 | 1.5 | 19 | 7.0 | 3.55 | 1.02 | 1.60 | 1.03 | 1.06 | 0.76 |
| **FIV-20** | 0 | 38 | 11.5 | 8.05 | 5.80 | 2.42 | 2.78 | 34 | 1.2 | 13 | 7.0 | 3.25 | 1.04 | 1.98 | 0.87 | 0.48 | 0.91 |
| **FIV-20** | 2 | 23 | 6.6 |  | 12.90 | 5.68 | 4.52 | 75 | 1.1 | 21 | 8.0 | 2.34 | 1.05 | 3.95 | 0.41 |  | 0.84 |
| **FIV-20** | 4 | 26 | 8.2 | 6.20 | 17.60 | 12.14 | 4.22 | 87 | 1.6 | 19 | 9.6 | 2.50 | 0.99 | 5.26 | 0.35 | 1.41 | 0.84 |
| **FIV-20** | 10 | 36 | 11.1 | 8.50 | 7.70 | 5.54 | 1.46 | 75 | 1.4 | 27 | 7.8 | 3.45 | 1.00 | 2.44 | 0.79 |  | 0.81 |
| **FIV-21** | 0 | 24 | 7.2 | 4.26 | 29.10 | 19.21 | 7.86 | 66 | 0.8 | 7 | 8.0 | 2.52 | 1.21 | 3.50 | 0.46 | 0.51 | 0.80 |
| **FIV-21** | 2 | 29 | 9.1 | 6.00 | 7.90 | 5.29 | 2.53 | 60 | 1.5 | 31 | 7.6 | 3.42 | 1.48 | 2.14 | 0.82 | 0.72 | 0.79 |
| **FIV-21** | 4 | 28 | 8.7 | 5.70 | 7.50 | 5.20 | 2.35 | 58 | 1.3 | 32 | 7.4 | 3.25 | 1.05 | 2.75 | 0.78 |  | 0.87 |
| **FIV-22** | 0 | 33 | 10.8 | 7.35 | 5.00 | 1.60 | 2.60 | 100 | 2.0 | 20 | 8.4 | 3.52 | 1.02 | 2.95 | 0.72 | 0.69 | 0.82 |
| **FIV-22** | 2 | 28 | 9.7 | 6.70 | 6.10 | 4.27 | 1.46 | 60 | 1.1 | 16 | 6.2 | 3.50 | 0.68 | 1.80 | 1.30 | 1.32 | 1.08 |
| **FIV-22** | 4 |  |  |  |  |  |  | 126 | 3.6 | 10 | 12.0 | 3.01 | 1.07 | 7.05 | 0.33 | 1.06 | 0.92 |
| **FIV-22** | 10 | 47 | 14.6 | 10.93 | 5.00 | 0.65 | 4.25 | 52 | 1.1 | 33 | 8.0 | 3.15 | 1.22 | 2.50 | 0.65 | 1.09 | 0.72 |
| **FIV-23** | 0 | 31 | 9.5 | 6.84 | 9.90 | 3.30 | 3.90 | 48 | 1.7 | 32 | 9.2 | 3.80 | 1.31 | 3.12 | 0.70 | 0.39 | 0.73 |
| **FIV-23** | 2 |  |  |  |  |  |  | 69 | 1.6 | 82 | 9.6 | 4.07 | 1.65 | 2.74 | 0.74 | 0.54 | 0.84 |
| **FIV-23** | 10 | 41 | 12.9 | 10.08 | 6.90 | 2.69 | 2.55 | 53 | 1.2 | 53 | 7.6 | 3.74 | 0.79 | 2.09 | 0.97 | 0.47 | 0.85 |
| **FIV-24** | 0 | 34 | 10.3 | 6.92 | 2.60 | 1.61 | 0.70 | 43 | 0.8 | 29 | 8.0 | 3.08 | 1.24 | 3.00 | 0.63 |  | 0.79 |
| **FIV-24** | 2 | 38 | 12.3 | 9.95 | 7.80 | 1.87 | 5.07 | 53 | 1.4 | 14 | 7.2 | 3.69 | 0.94 | 1.92 | 1.05 | 0.78 | 0.86 |
| **FIV-24** | 4 | 50 | 14.3 | 8.75 | 18.60 | 5.60 | 8.90 | 57 | 1.3 | 36 | 7.4 | 3.33 | 1.03 | 2.37 | 0.82 | 3.08 | 0.89 |
| **FIV-24** | 10 | 24 | 7.5 | 6.83 | 25.60 | 17.66 | 4.35 | 67 | 1.1 | 92 | 9.4 | 3.61 | 1.47 | 3.18 | 0.62 | 1.33 | 0.87 |
| **FIV-25** | 0 | 38 | 12.8 | 7.38 | 6.00 | 2.76 | 2.88 | 52 | 1.5 | 33 | 9.0 | 3.63 | 1.29 | 3.16 | 0.68 | 1.18 | 0.78 |
| **FIV-25** | 4 | 36 | 10.7 | 9.41 | 15.40 | 10.47 | 4.62 |  |  |  |  |  |  |  |  |  | 0.82 |
| **FIV-25** | 10 | 22 | 7 | 6.80 | 17.10 | 10.43 | 3.59 | 88 | 1.9 | 116 | 11.8 | 5.14 | 1.91 | 3.29 | 0.77 | 0.71 | 0.92 |
| **FIV-26** | 0 | 29 | 10.7 | 7.64 | 5.40 | 2.97 | 2.00 | 59 | 1.0 | 33 | 8.2 | 3.54 | 1.35 | 2.93 | 0.76 | 1.33 | 0.82 |
| **FIV-26** | 2 | 38 | 11.5 | 5.46 | 21.30 | 9.30 | 10.70 | 36 | 1.3 | 15 | 8.3 | 2.55 | 0.76 | 3.25 | 0.44 |  | 0.99 |
| **FIV-26** | 4 | 31 | 9 | 5.32 | 22.40 | 8.70 | 12.70 | 58 | 1.5 | 28 | 9.5 | 3.25 | 1.35 | 4.24 | 0.52 | 1.49 | 1.00 |
| **FIV-27** | 0 | 54 | 16.2 | 8.33 | 19.50 | 15.30 | 2.80 | 62 | 1.2 | 15 | 9.6 | 3.01 | 1.23 | 4.73 | 0.46 |  | 0.76 |
| **FIV-27** | 2 | 24 | 6.4 | 8.08 | 15.80 | 12.30 | 2.10 |  |  |  | 9.8 | 3.20 | 1.05 | 5.54 | 0.47 | 1.54 | 0.76 |
| **FIV-27** | 4 | 30 | 9 | 5.45 | 20.40 | 15.30 | 4.08 | 93 | 1.5 | 22 | 10.6 | 3.70 | 0.83 | 5.98 | 0.53 | 1.68 | 1.04 |
| **FIV-27** | 10 | 38 | 11.9 | 7.77 | 7.60 | 3.50 | 3.80 | 59 | 1.2 | 42 | 6.8 | 3.75 | 0.82 | 1.19 | 1.23 | 2.26 | 0.86 |
| **FIV-28** | 0 | 35 | 10.8 | 6.93 | 10.90 | 5.34 | 5.34 | 28 | 1.2 | 18 | 6.0 | 3.06 | 0.85 | 1.44 | 1.04 | 1.05 | 0.77 |
| **FIV-28** | 2 | 37 | 11 | 7.95 | 7.70 | 4.47 | 2.77 | 107 | 1.1 | 25 | 10.4 | 4.50 | 1.61 | 3.65 | 0.71 | 1.32 | 1.13 |
| **FIV-28** | 4 | 31 | 8.9 | 5.46 | 24.00 | 7.20 | 10.70 | 78 | 2.5 | 15 | 9.8 | 2.55 | 0.81 | 6.07 | 0.35 |  | 1.09 |
| **FIV-28** | 10 | 39 | 11.8 | 7.75 | 6.40 | 3.26 | 2.88 | 61 | 1.5 | 34 | 6.8 | 3.54 | 1.24 | 1.41 | 1.09 | 1.97 | 0.99 |
| **FIV-29** | 0 | 25 | 7.5 | 4.99 | 8.40 | 1.26 | 4.79 | 128 | 1.8 | 27 | 8.4 | 3.52 | 1.27 | 3.23 | 0.73 | 0.86 | 0.94 |
| **FIV-29** | 2 | 36 | 12 | 9.37 | 11.90 | 9.76 | 1.90 | 44 | 1.1 | 13 | 9.6 | 3.28 | 0.98 | 4.36 | 0.57 |  | 1.07 |
| **FIV-29** | 10 | 26 | 8.4 | 5.04 | 7.14 |  |  | 24 | 2.3 | 35 | 10.7 | 3.36 | 1.15 | 5.21 | 0.46 | 1.47 | 0.95 |
| **FIV-30** | 0 | 25 | 7.9 | 5.85 | 7.90 | 4.27 | 3.63 | 20 | 0.6 | 6 | 6.6 | 2.61 | 1.23 | 1.96 | 0.65 | 0.71 | 0.81 |
| **FIV-30** | 4 | 25 | 7.6 | 6.10 | 16.40 | 12.63 | 3.77 | 47 | 0.9 | 11 | 9.6 | 2.80 | 1.32 | 4.59 | 0.41 | 0.92 | 0.87 |
| **FIV-30** | 10 | 28 | 8.7 | 6.03 | 18.60 | 8.56 | 8.93 | 44 | 1.1 | 13 | 8.6 | 3.22 | 1.22 | 3.22 | 0.60 | 0.37 | 0.99 |
| **FIV-31** | 0 | 23 | 7.9 | 5.95 | 9.00 | 6.70 | 1.35 | 75 | 1.3 | 19 | 7.0 | 3.53 | 1.12 | 1.53 | 1.02 | 1.54 | 1.12 |
| **FIV-31** | 2 | 30 | 9.6 |  | 10.00 | 6.80 | 2.90 | 43 | 1.1 | 12 | 8.4 | 3.16 | 1.22 | 3.38 | 0.60 | 1.01 | 0.97 |
| **FIV-31** | 4 | 32 | 10.8 | 6.90 | 10.90 | 5.44 | 3.50 | 38 | 1.2 | 18 | 8.2 | 3.18 | 1.10 | 3.35 | 0.63 | 1.54 | 0.92 |
